# Sensors Based on Metal Nanoclusters Stabilized on Designed Proteins

**DOI:** 10.3390/bios8040110

**Published:** 2018-11-15

**Authors:** Antonio Aires, Elena Lopez-Martinez, Aitziber L. Cortajarena

**Affiliations:** 1 CIC biomaGUNE, Parque Tecnológico de San Sebastián, Paseo Miramón 182, 20014 San Sebastián, Spain; aaires@cicbiomagune.es (A.A.); elopez@cicbiomagune.es (E.L.-M.); 2 Ikerbasque, Basque Foundation for Science, Mª Díaz de Haro 3, 48013 Bilbao, Spain

**Keywords:** protein design, metal nanocluster, fluorescent probe, nanosensor, temperature sensor, metal sensors, ROS sensors

## Abstract

Among all new nanomaterials, metal nanoclusters (NCs) have attracted special attention due to their interesting optical properties, among others. Metal NCs have been recently studied and used as sensors for different analytes. However, there is a need to explore the potential of these new sensors in a systematic manner and to develop new systems to broaden the possibilities that sensing offers to the industry. In this work, we show the potential use of repeat protein scaffolds as versatile templates for the synthesis and stabilization of various metal NCs, specifically Au, Ag, and CuNCs. The resulting protein-metal NCs hybrids are evaluated as sensors for different stimuli such as temperature, ions, or reactive oxygen species (ROS). Among the three protein-metal NCs, all performed nicely as temperature sensors, AuNCs responded to metal ions, and AgNCs were able to detect ROS.

## 1. Introduction

Metal nanostructures are one of the most developed and studied systems in nanotechnology over the past decades, especially metal nanoparticles (NPs) and nanoclusters (NCs), due to their optical, magnetic, and catalytic properties. In particular, metal NCs smaller than 2 nm display different fluorescence emissions depending on their size, and also the molecules used for their stabilization [1]. The interesting optical properties that NCs feature are owed to their small size, comparable to Fermi wavelength of electrons, transforming the electrons from the performance of metal bulk into a quantum behavior, with discrete levels of energy. Fluorescent metal NCs have been widely used as sensors due to their response to certain changes in their medium such as temperature [2,3,4,5,6,7], oxidative stress, acidity, or the presence of different substances such as metal ions [8,9,10]. Metal NCs are usually synthetized using capping agents or proteins such as bovine serum albumin (BSA) as templates, although this approach does not give a precise control over the number of clusters per protein molecule or their position.

Protein engineering can be used for designing functionalized tools that fulfill the desired requirements. Simple modular proteins are excellent to use as templates for designing functional nanostructures because of their stability properties, modularity, and structure. For example, in a consensus tetratricopeptide repeat (CTPR), derived from natural tetratricopeptide repeat (TPR) sequences, only eight residues of the 34 within each CTPR unit are conserved and have structural significance, leaving plenty of space for mutations and changes alongside the protein. In the past, we showed an easy way to template AuNCs using CTPR proteins [11], and used this system to build a fluorescent probe for the recognition of a TPR-binding peptide. In the present work, we explore the repeat protein scaffolds as versatile templates for the synthesis and stabilization of different metal NCs and their potential use for sensing different stimuli, such as temperature, ions, and reactive oxygen species (ROS), with the main objective of developing an array modular sensing tools that could be combined in the same protein scaffold.

## 2. Materials and Methods

### 2.1. Chemicals

All chemicals were purchased from Sigma–Aldrich and used without further purification. Ultrapure reagent grade water (18.2 MΩ, Wasserlab, Navarra, Spain) was used in all experiments.

### 2.2. Purification of CTPR Protein

The CTPR protein was produced following standard molecular biology protocols for recombinant protein expression. The CTPR3_cys protein cloned into pPro-EX-HTb vector were transformed in *Escherichia coli* C41 (DE3) cells. The cells were grown in Luria–Bertani media (LB) with ampicillin. The protein expression was induced by isopropyl β-d-1-thiogalactopyranoside (IPTG) at optical density of 0.6–0.8. After 16 h expression at 20 °C, cells were harvested, and the his-tagged proteins were purified via affinity chromatography using Ni-NTA resin. The his-tag was cleaved using Tobacco Etching Virus (TEV) protease, and a second Ni-NTA affinity column purification was performed in order to remove the his-tag and the TEV protease from the protein sample. The protein concentration was determined by absorbance at 280 nm using the extinction coefficient calculated from the amino acid composition. The CTPR3 with an additional cysteine residue at the C-terminal (C3_cys) has the following amino acid sequence:

GAMDPGNSAEAWYNLGNAYYKQGDYDEAIEYYQKALELDPNNAEAWYNLGNAYYKQGDYDEAIEYYQKALELDPNNAEAWYNLGNAYYKQGDYDEAIEYYQKALELDPNNAEAKQNLGNAKQKQGC (Molecular weight: 14463.4 Daltons).

### 2.3. Synthesis and Characterization of Protein-Stabilized Metal Nanoclusters

The protein-stabilized metal NCs were synthesized following a previously reported procedure using sodium ascorbate instead of ascorbic acid to provide the reducing environment [11]. Briefly, 1000 µL of protein at 10 µM were mixed with HAuCl_4_, AgNO_3_ or CuSO_4_ (50 µL 10 mM, 50 equation respect to protein) for at least 30 min to allow the adsorption of metal ions to the protein’s stabilizing sites. Then, the reduction of the metal ions to metal NCs was achieved by adding 50 µL of sodium ascorbate at 100 mM (10 equation respect to metal ions). The reaction was incubated at 37 °C for 24 h. The samples were concentrated to 500 μL using Amicon ultrafiltration tubes with a 10-kDa membrane. Finally, the unreacted salts were removed by gel filtration chromatography, using an Illustra NAP-25 column equilibrated with phosphate-buffered saline (PBS). The purified samples were concentrated using Amicon 10 kDa ultrafiltration tubes until 40 µM and kept at 4 °C for further experiments. The samples were diluted in PBS when necessary for the experiments. The absorption and fluorescence spectra of protein stabilized metal NCs were recorded using a spectrophotometer UV-Vis (Jasco V630-Bio) and a fluorometer (Perkin Elmer LS55), respectively.

### 2.4. Fluorescence Quantum Yield

The fluorescence quantum yield (Φ_x_) was calculated using anthracene in ethanol as a reference (Φ_ref_ = 0.27, λ_exc_ = 370 nm and λ_em_ = 423 nm) and the following formula:

(1) $$\phi_{x}=\phi_{\mathrm{ref}}\frac{{\mathrm{Grad}}_{x}}{{\mathrm{Grad}}_{\mathrm{ref}}}\left(\frac{\eta_{x}{}^{2}}{\eta_{\mathrm{ref}}{}^{2}}\right)$$

where Grad_x_ and Grad_ref_ are the gradient from the plot of integrated fluorescence intensity versus absorbance at excitation wavelength, for the sample and the reference, respectively, and η_x_ and η_ref_ are the refractive indexes of the solvents, water, and ethanol, respectively. Fluorescence measurements were performed using a fluorometer (Perkin Elmer LS55), and absorbance was recorded with a spectrophotometer UV-Vis (Jasco V630-Bio) in quartz cuvettes.

### 2.5. Matrix Assisted Laser Desorption Ionization—Time Of Flight (MALDI-TOF) Mass Spectrometry

Mass spectra were acquired on an Applied Biosystems Voyager Elite MALDI-TOF mass spectrometer with delayed extraction (Applied Biosystems, Framingham, MA, USA) equipped with a pulsed N2 laser (λ = 337 nm). Sinapic acid was used as matrix. An extraction voltage of 20 kV was used. All mass spectra were acquired in positive reflection mode using delayed extraction with an average of 50–100 laser shots. MALDI-TOF sample preparation included 1 μL of the sample at 20 μM mixed with 4 μL of sinapic acid in 50:50 water/acetonitrile with 0.01% trifluoroacetic acid (TFA). Then, 1 μL of the mixture was deposited onto the MALDI plate and allowed to air-dry. The instrument was externally calibrated using monoisotopic peaks from the sinapic acid matrix (MH+ at *m*/*z* 225.071).

### 2.6. Temperature Sensing

The fluorescence spectra of a C3_cys-metal NCs suspension at 10 µM of protein concentration were measured at different temperatures ranging from 25 °C to 65 °C. The reversibility and the cycle stability of the metal NCs as temperature sensors were tested repeating the process for 5 cycles.

### 2.7. Ion Detection

To evaluate the selectivity of the protein-stabilized metal NCs towards several ion species, different ions at 10 μM including Na^+^, K^+^, Ag^+^, Ca^2+^, Ba^2+^, Cd^2+^, Co^2+^, Pb^2+^, Zn^2+^, Ni^2+^, Mn^2+^, Mg^2+^, Fe^2+^, Fe^3+^, and Hg^2+^ were incubated with the metal NCs. Briefly, 500 µL of the protein-stabilized metal NCs at 10 µM were mixed with 5 µL of the different ion solutions at 1 mM. After 30 min of reaction, 200 μL of the reactant solution was transferred into a quartz cuvette for fluorescence spectra recording at room temperature.

Copper detection was evaluated by the incubation of protein-stabilized metal NCs in phosphate-buffered solution (10 mM phosphate pH 7.4) with ion solutions at different concentrations (0–10 μM). Briefly, 5 µL of Cu^2+^ solutions of different concentrations (0–10 μM) obtained by serial dilution of the stock solution (10 mM) were added to 500 µL of the protein-stabilized metal NCs at 10 µM. After 30 min of incubation, 200 μL of the solution were transferred into a quartz cuvette and the fluorescence spectra recorded at room temperature.

### 2.8. ROS Detection

For the detection of reactive oxygen species (ROS), the assays were performed using Rose Bengal as the synthesizer for ROS. This dye, when irradiated with green light, is able to produce singlet oxygen molecules. As a control for the presence of ROS species 2,2′-azino-bis(3-ethylbenzthiazoline-6-sulphonic acid) (ABTS) was used. 2,2′-azino-bis(3-ethylbenzthiazoline-6-sulphonic acid) is widely used in antioxidants studies as the reporter for oxidative stress environment [12,13]. In a typical experiment, 90 µL of protein stabilized metal NCs at 40 μM in phosphate-buffered solution were mixed with 10 µL of Rose Bengal at 100 mM. The fluorescence was measured in a quartz cuvette before and after the irradiation with a green lamp during 15 min steps for a total time of 60 min. In parallel, 25 µL of ABTS were mixed with 10 µL of Rose Bengal (100 mM) and 65 µL of phosphate-buffered solution as a positive control of ROS detection. Absorbance was measured using a Jasco spectrophotometer (model V630BIO UV-Vis) before and after irradiation with a green LED lamp during 15 min steps for a total time of 60 min.

## 3. Results

### 3.1. Synthesis and Characterization of Protein-Stabilized Metal Nanoclusters

**Figure 1:**
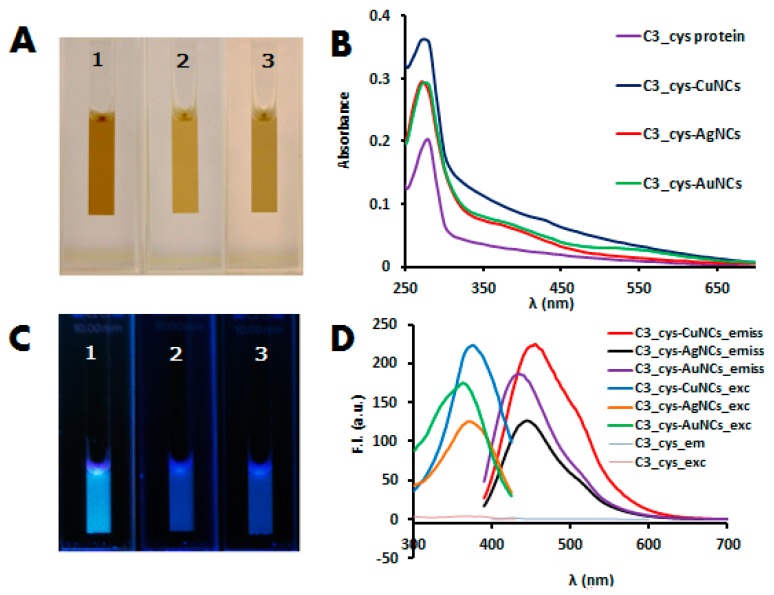
(**A**,**C**) images of consensus tetratricopeptide repeat (CTPR)-metal nanoclusters (NCs) solution under visible light and UV light: C3_cys-CuNCs (1), C3_cys-AgNCs (2), and C3_cys-AuNCs (3). (**B**) Absorption spectra of the non-conjugated CTPR protein and the synthetized protein-NCs conjugates. (**D**) Excitation and emission spectra of CTPR control protein without NCs and CTPR templated NCs.

Blue fluorescent protein stabilized metal NCs were synthesized in one step by reducing the metal salt (HAuCl_4_, AgNO_3_ or CuSO_4_) with sodium ascorbate in the presence of C3_cys protein at 37 °C for 72 h. The as-obtained protein stabilized metal NCs suspension are light brown under visible light (Figure 1A) and emit strong blue fluorescence under 365 nm irradiation (Figure 1B). The UV-visible spectra (Figure 1C) of the protein-stabilized metal compared with the spectrum of the protein, at the same concentration, showed in addition to the characteristic protein absorption at 280 nm the presence of small and broad peaks around 350–370 nm and in the case of AuNCs a small peak around 560 nm due to the presence of a small fraction of gold nanoparticles. The fluorescent protein-stabilized metal NCs showed maximum excitation and emission peaks at 375 and 453 nm (CuNCs), 371 and 445 nm (AgNCs), and 365 and 438 nm (AuNCs), respectively; while the protein without metal NCs did not produce any fluorescence signal (Figure 1D).

The fluorescence quantum yield (Φ_x_) of C3_cys-CuNCs, C3_cys-AgNCs, and C3_cys-AuNCs were 4.1, 2.6, and 3.5%, respectively, when anthracene was used as a reference. These values are in the same range as the ones reported for other protein-stabilized fluorescent metal NCs in the literature [14,15,16].

**Figure 2:**
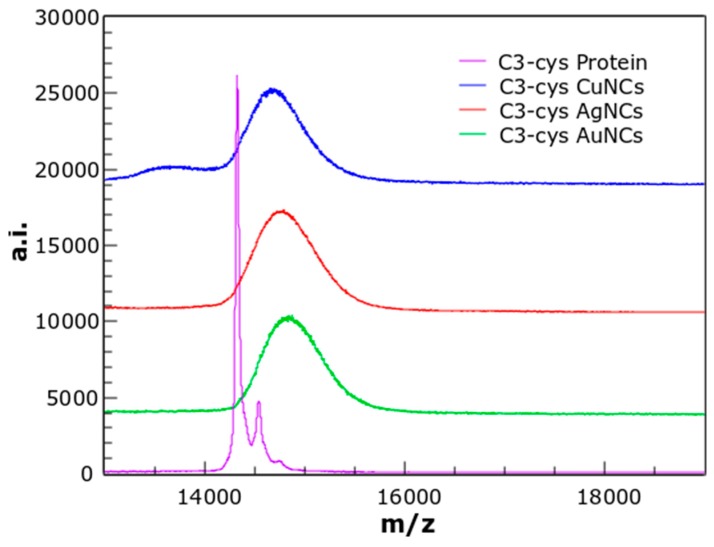
Mass spectra of non-conjugated C3_cys protein (magenta trace), and the protein-NCs conjugates: C3_cys-CuNCs (blue trace), C3_cys-AgNCs (red trace), and C3_cys-AuNCs (green trace). The shifts in the peaks relative to the protein spectrum correspond with the absorption of six Cu atoms, five Ag atoms, and three Au atoms.

MALDI-TOF mass spectrometry was used to determine the size of the NCs. The protein-NCs complexes spectra showed clear shifts compared to the protein spectrum. However, the peaks were wider than the pure protein peak, indicating the presence of populations of protein with different numbers of metal atoms. Thus, it is possible that the laser irradiation during the MALDI-TOF acquisition was etching the NCs. The mass spectrum of C3_cys-CuNCs (Figure 2) showed a main peak at *m*/*z* = 14,693.34 Da that corresponds to C3_cys-CuNCs with six copper atoms per protein (compared to a *m*/*z* = 14,321.65 Da for free protein). Similarly, the mass spectrum of C3_cys-AuNCs showed a peak at *m*/*z* = 14,869.47 Da indicating three gold atoms per protein, and the mass spectrum of C3_cys-AgNCs showed a peak at *m*/*z* = 14,789.94 Da that corresponds to five silver atoms per protein. It is plausible that other NCs species were also formed; however, they were not clearly detected by MALDI-TOF mass spectrometry.

### 3.2. Temperature Sensing

**Figure 3:**
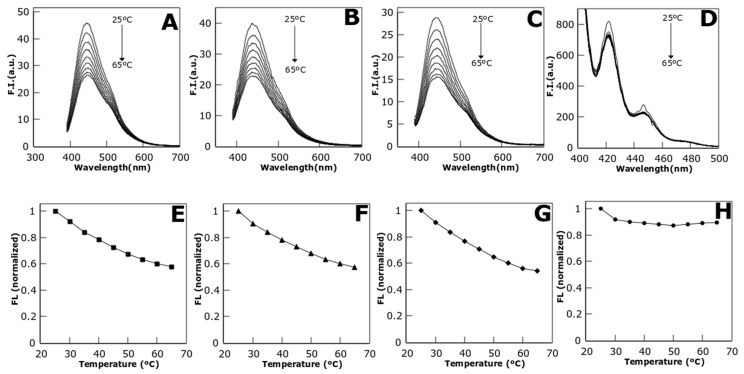
CTPR-templated metal NCs emission spectra under a temperature gradient from 25 °C to 65 °C (upper row) for C3_cys-CuNCs (**A**); C3_cys-AgNCs (**B**); C3_cys-AuNCs (**C**); and anthracene, as reference molecule (**D**). Normalized fluorescence intensity vs. temperature (lower row) for C3_cys-CuNCs (**E**); C3_cys-AgNCs (**F**); C3_cys-AuNCs (**G**); and anthracene (**H**).

Protein stabilized metal NCs fluorescence was tested under a temperature range from 25 °C to 65 °C (Figure 3). The fluorescence decays up to 42.47% in the case of CuNCs, 45.84% for AgNCs, and 42.85% in the case of AuNCs (Figure 3E–G respectively). Comparatively, the effect of the temperature on the fluorescence of a control organic molecule, anthracene, is smaller (Figure 3D,H), showing a fluorescence quenching of 10.5% at the maximum temperature tested. This difference empowers the potential use of CTPR templated NCs as thermos-sensing devices due to their more linear response to temperature changes than some dyes as anthracene, although other organic dyes, such as Rhodamine C present also a linear response to temperature [17]. However, CTPR templated NCs present the advantage of being water-soluble and fluorescent in physiological conditions.

Potential hysteresis effects may hamper the use of a sensor as a reliable nanothermometer, thus, it is important to evaluate that the fluorescence intensity at a certain temperature is not dependent on the heating or cooling cycles and remains the same when the system is heating up or is cooling down. Hysteresis effects may emerge from local conformational changes of the protein at the surroundings of the metal NC [4]. Nevertheless, CTPR proteins present a good thermodynamic stability under the temperature range in which the sensor was evaluated [18,19] and a fully reversible thermal denaturation [20], therefore these scaffolds guarantee a system without hysteresis.

**Figure 4:**
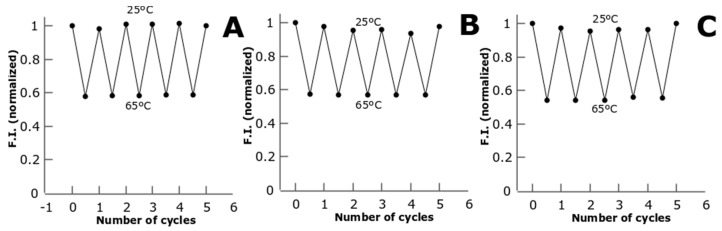
Normalized fluorescence intensity vs. the number of temperature cycles between 25 °C and 65 °C for C3_cys-CuNCs (**A**), C3_cys-AgNCs (**B**), and C3_cys-AuNCs (**C**).

We also tested the stability of the fluorescent signal from the metal NCs through different cycles of temperature. We observe that the metal NCs can undergo several cycles of heating and cooling from 25 °C to 65 °C without losing the temperature response and the resolution of the detection (Figure 4). The fact that CTPR-templated metal NCs can perform several cycles without losing fluorescence or temperature sensitivity makes them robust temperature sensors.

### 3.3. Ion Detection

**Figure 5:**
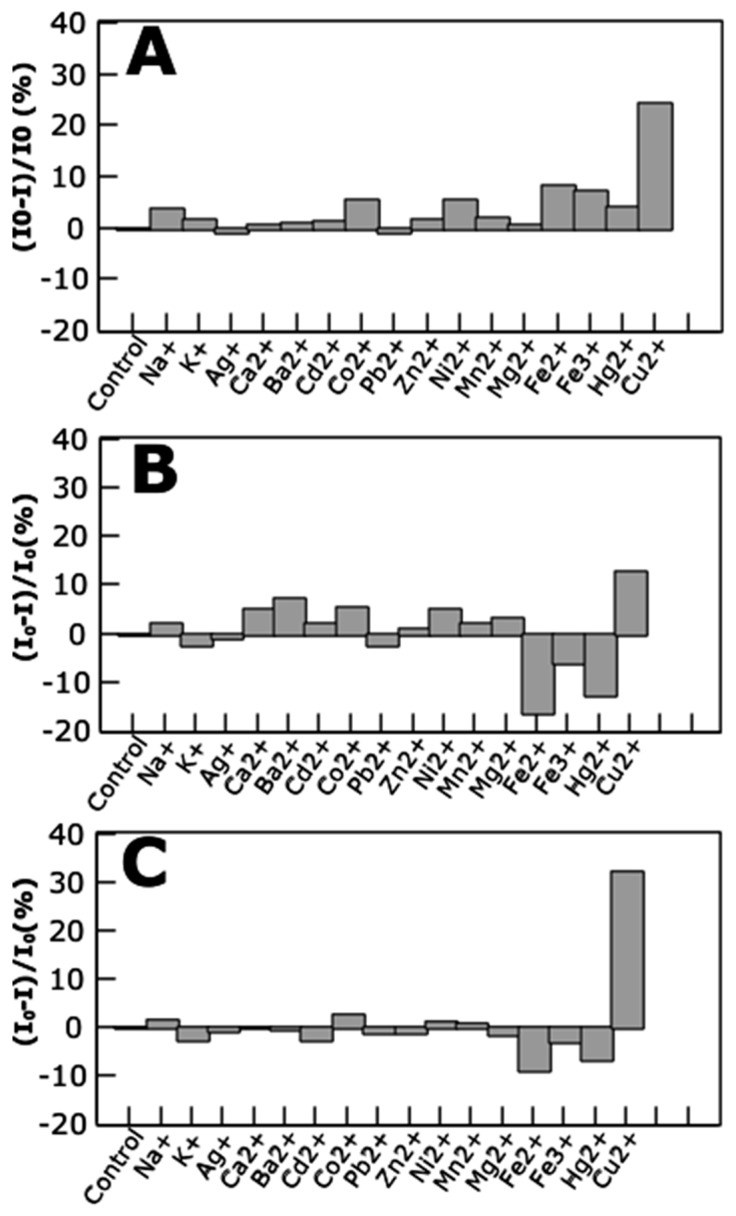
Ion detection by protein-stabilized NCs. The change in the fluorescence emission intensity of the protein-stabilized nanoclusters upon addition of different ions. C3_cys-CuNCs (**A**); C3_cys-AgNCs (**B**); and C3_cys-AuNCs (**C**).

Several ions were tested as targets for the sensing properties of the protein-stabilized metal NCs. Among these ions, copper performed as the strongest quencher of the fluorescence emission of the metal NCs, for each of the NCs composition (Figure 5). Besides, AgNCs and AuNCs showed an enhanced fluorescence in response to the presence of Fe^2+^ and Hg^2+^, while this phenomenon is not observed for the CuNCs. Interestingly, other works have reported a quenching in fluorescence from AuNCs [21] and CuNCs [22] triggered by the presence of Hg^2+^ ions.

**Figure 6:**
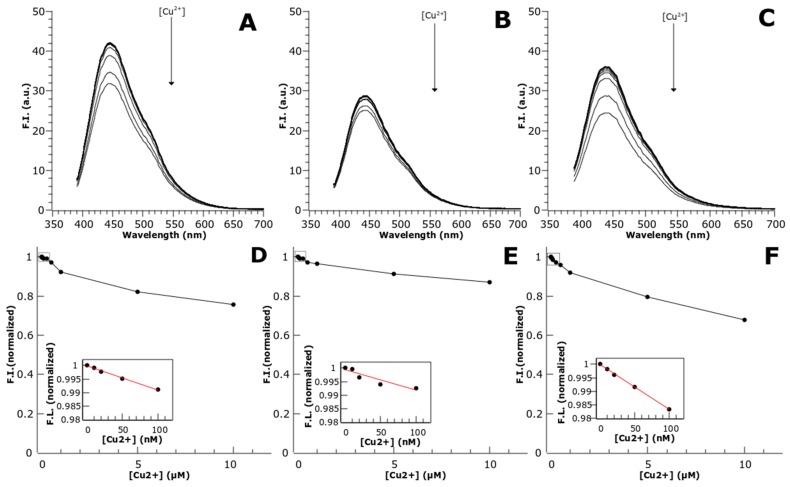
Cu detection by protein-stabilized nanoclusters. Fluorescence emission spectra of CTPR templated metal NCs in the presence of different Cu^2+^ concentrations (upper panels) C3_cys-CuNCs (**A**); C3_cys-AgNCs (**B**); and C3_cys-AuNCs (**C**). Normalized fluorescence vs. Cu^2+^ concentration plots (lower panels). C3_cys-CuNCs (**D**); C3_cys-AgNCs (**E**); and C3_cys-AuNCs (**F**).

Moreover, the protein-stabilized AuNCs were the most responsive to Cu^2+^ ions, being able to quench up to 20.4% of the fluorescence at 5 µM of Cu^2+^ and 32.31% of the fluorescence at 10 µM of Cu^2+^ (Figure 6). This phenomena has already been reported in previous works using BSA-AuNCs [8]; however, this work showed Cu^2+^ detection at mM concentration with only a 10% of fluorescence quenching at 10 µM Cu^2+^; whereas the CTPR templated AuNCs were sensitive to nanomolar concentrations Cu^2+^ and showed at 10 µM Cu^2+^ a quenching three times stronger.

No studies have been performed to explore the mechanism of fluorescence quenching by copper, but there are some possible explanations of this effect. The linear response of the fluorescence quenching to the [Cu^2+^] for CuNCs and AuNCs, for which a [Cu^2+^] of 0.1 µM added to 10 µM of NCs (1% analyte relative to the sensor) results in a fluorescence decrease of approximately 1%, supports a Langmuir-type adsorption behavior for the Cu^2+^ ions. The mechanism of quenching could be due to the paramagnetic nature of Cu^2+^, which can enhance intersystem crossing (ISC) as a way of non-radiative dissipation of the energy absorbed, competing with fluorescence emission, and thus quenching the fluorescence of the metal NCs [23]. Another possible explanation is that there is some de-attachment of AuNCs from the coordination sites in the protein due to a reaction with Cu^2+^, thus leading to a loss of fluorescence. Further characterization of this sensing behavior would be necessary in order to detangle the molecular mechanism underlying fluorescence quenching by copper.

### 3.4. Reactive Oxygen Species (ROS) Detection

**Figure 7:**
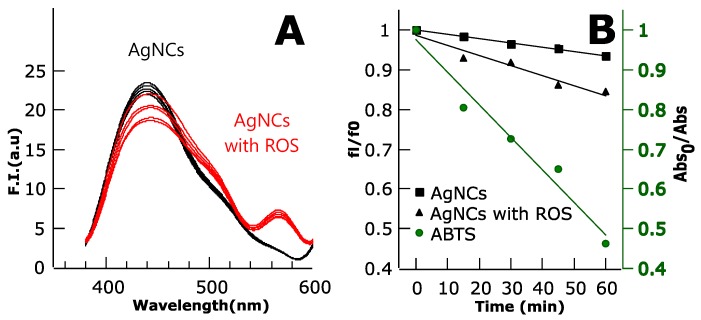
(**A**) Fluorescence emission spectra of the C3_cys AgNCs. Black lines represent no reactive oxygen species (ROS) control samples and red lines correspond to Rose Bengal-metal NCs samples, i.e., with oxidative stress from ROS production. (**B**) Stern–Volmer plot showing the quenching of the AgNCs fluorescence with time upon irradiation of Rose Bengal (RB), related to increased amounts of ROS. ABTS was used as a probe for ROS production; its absorbance was measured to check the formation of ROS (green spheres, absorbance in green axis).

The different NCs samples were evaluated as sensors for the detection of oxidative stress through the presence of reactive oxygen species in the environment. The Ag-based NCs were the only protein-templated NCs that showed reliable response to ROS (Figure 7). There was a quenching in the fluorescence of the AgNCs in response to the amount of ROS produced by RB in time. The quenching occurred slowly in time, with a 10% of fluorescence loss in an hour. It is important to note that the positive control used to detect ROS formation, ABTS, showed larger response to the presence of ROS than the AgNCs.

In the case of the copper nanocluster, an initial quenching of the system before sample irradiation was observed (Figure 7A), as well as some photobleaching as seen in the control sample of the protein without ROS (Figure 7B). However, there was no further quenching upon irradiation when ROS were being synthetized (data not shown). It is possible that Rose Bengal was quenching the NCs fluorescence due to some interaction between the molecule and the Ag atoms. The mechanism on how ROS quenches the fluorescence of AgNCs was not yet solved in our system; however, it is possible that oxido-reduction changes on the AgNCs directed by ROS activity induced fluorescence quenching, as has been reported before [24,25]. In the case of the protein-templated AuNCs, the absence of response to ROS is supported by the fact that AuNCs have good redox stability, whereas in the case of the protein templated CuNCs the lack of response to ROS is not explained by their redox stability, since they are less stable than AgNCs.

## 4. Conclusions

In previous work we developed new technologies in order to use designed CTPR proteins for the controlled growth of AuNCs. The main objective of the current work was to evaluate the potential of repeat protein scaffolds as versatile templates for the synthesis and stabilization of different metal NCs (Au, Ag, and CuNCs) to explore the use of CTPR stabilized metal NCs as sensors for different conditions, such as temperature, ions, or ROS. We want to highlight the sensitivity that these NCs have for the temperature sensing and the ability to undergo several temperature cycles without losing sensing efficiency. Moreover, AuNCs can be used to detect small concentrations of copper ions in solution and AgNCs showed sensitivity to ROS species. Although there are previously reported systems that showed similar detection capabilities, and in some cases with better specificity and detection limits, the major advantage in CTPR templated metal NCs is that the same system can encode different sensing capabilities and functionalities, by just changing the metal attached. Therefore, this work demonstrates the versatility of the repeat protein scaffolds to obtain protein-stabilized nanoclusters with different metal composition, which increases their potential use in the field of biosensing. Due to the modular nature of these proteins, we envision the potential of CTPR protein templated metal NCs as integrated modules in larger assemblies of CTPR proteins. Using their modularity and with different functional elements alongside the protein array, we could achieve the building of multi-component sensing tools.

## 5. Patents

Patent: A.A. and A.L.C. Metal Nanocluster Scaffolds. European patent (EP17382451.7), PCT application (PCT/EP2018/068710).
